# Intermittent Use of a Short-Course Glucagon-like Peptide-1 Receptor Agonist Therapy Limits Adverse Cardiac Remodeling via Parkin-dependent Mitochondrial Turnover

**DOI:** 10.1038/s41598-020-64924-2

**Published:** 2020-05-19

**Authors:** Juliana de F. Germano, Chengqun Huang, Jon Sin, Yang Song, Kyle C. Tucker, David J. R. Taylor, Hannaneh Saadaeijahromi, Aleksandr Stotland, Honit Piplani, Roberta A. Gottlieb, Robert M. Mentzer, Allen M. Andres

**Affiliations:** 1 Cedars Sinai Medical Center, Smidt Heart Institute, Los Angeles, USA; 2 University of California, San Diego, Department of Medicine, San Diego, USA

**Keywords:** Cardiology, Cardiovascular biology

## Abstract

Given that adverse remodeling is the leading cause of heart failure and death in the USA, there is an urgent unmet need to develop new methods in dealing with this devastating disease. Here we evaluated the efficacy of a short-course glucagon-like peptide-1 receptor agonist therapy—specifically 2-quinoxalinamine, 6,7-dichloro-N-(1,1-dimethylethyl)-3-(methylsulfonyl)-,6,7-dichloro-2-methylsulfonyl-3-N-tert-butylaminoquinoxaline (DMB; aka Compound 2) – in attenuating adverse LV remodeling. We also examined the role, if any, of mitochondrial turnover in this process. Wild-type, Parkin knockout and MitoTimer-expressing mice were subjected to permanent coronary artery ligation, then treated briefly with DMB. LV remodeling and cardiac function were assessed by histology and echocardiography. Autophagy and mitophagy markers were examined by western blot and mitochondrial biogenesis was inferred from MitoTimer protein fluorescence and qPCR. We found that DMB given post-infarction significantly reduced adverse LV remodeling and the decline of cardiac function. This paralleled an increase in autophagy, mitophagy and mitochondrial biogenesis. The salutary effects of the drug were lost in Parkin knockout mice, implicating Parkin-mediated mitophagy as part of its mechanism of action. Our findings suggest that enhancing Parkin-associated mitophagy and mitochondrial biogenesis after infarction is a viable target for therapeutic mitigation of adverse remodeling.

## Introduction

Adverse post-infarction (post-MI) LV remodeling is the most common cause of heart failure in the USA^1^. More than 6.5 million Americans suffer from HF and the high 5-year mortality rate is associated with a healthcare cost of approximately $39 billion/year^2^. While angiotensin-converting enzyme inhibitors, angiotensin II receptor blockers, and beta blockers are the mainstay of pharmacologic therapy directed at limiting adverse remodeling, the benefits are most often seen in patients with large infarcts and individuals who are not candidates for reperfusion therapies. Moreover, their use is associated with no more than a 20–25% reduction in major adverse cardiac events^3^. Apart from a drug that is a combination of valsartan (an AngII receptor blocker) and sacubitril (a neprilysin inhibitor) there have been no new treatments introduced clinically for the past three decades that specifically target adverse post-MI LV remodeling^4^. One approach may lie with development of a better understanding of the effect of brief treatment with a glucagon like peptide-1 receptor (GLP1R) agonist on mitophagy and mitochondrial biogenesis when administered after the MI and the potential role of mitochondrial quality control in mitigating adverse remodeling and the development of heart failure. GLP1R agonists represent a unique class of hypoglycemic drugs that are known to be effective in the treatment of Type 2 Diabetes Mellitus^5–8^. There is also clinical evidence that suggest these agonists are effective in reducing the incidence of major adverse cardiovascular events^9–11^. Specifically, they have been shown to reduce cardiovascular mortality, non-fatal myocardial infarction, non-fatal stroke, and all-cause mortality risk.

The naturally occurring GLP1R agonist is glucagon like peptide-1 which is released by intestinal L-cells in response to feeding and stimulates pancreatic release of insulin^12,13^. It has a short half-life in circulation ranging from 1–5 minutes and is rapidly degraded by dipeptidyl peptidase-IV^14–17^. Longer lasting GLP1R agonists such as liraglutide and exenatide were developed to be resistant to this degradation and are effective in treating type 2 diabetes mellitus^5–8^. However, their chronic use is associated with increased risk of hypoglycemia, abdominal pain, pancreatitis, pancreatic cancer and thyroid cancer^18,19^. The GLP1R agonist 6,7-dichloro-2-methylsulfonyl-3-N-tert-butylaminoquinoxaline (DMB, also known as compound 2) is a unique quinoxaline-based small-molecule agonist of GLP1Rs first identified by Knudsen and colleagues. It is a potent agonist of GLP1Rs. It is an ago-allosteric agonist of GLP1Rs that also potentiates GLP-1 binding to GLP1Rs^20–22^. Since it does not bind to the canonical orthosteric binding region utilized by GLP-1 or other GLP1R agonists, it is resistant to known GLP1R antagonists such as exendin (9–39) amide and JANT-4^22,23^. We chose to use DMB administered 3×/week for just 2 weeks based on the expectation that (1) short-course intermittent therapy would be less likely to cause systemic complications such as hypoglycemia or pancreatitis; and (2) that intermittent induction of autophagy and mitophagy might allow time to restore mitochondrial content through mito-biogenesis.

While the molecular mechanism(s) underlying the cardioprotective effects of this class of drugs have yet to be identified, there are preclinical studies that suggest GLP1R agonists may also be beneficial when administered shortly after myocardial infarction and that the underlying mechanism might be due to the activation of mitophagy. DeNicola *et al*. (2014) reported that mice subjected to permanent coronary artery ligation and administered daily injections of the antidiabetic drug exendin-4 exhibited less myocardial hypertrophy, reduced interstitial fibrosis, and increased survival^24^. Based on studies in H9C2 cardiomyoblasts, they concluded this effect was mediated in part by suppression of the production of reactive oxygen species and the opening of the mitochondrial permeability transition pore. Qiao *et al*. (2018) explored the possibility that the antidiabetic drug liraglutide would protect against cardiomyocyte death in the infarcted heart^25^. They found that rats treated with liraglutide for 4 weeks after the infarct were associated with a reduction in cardiac fibrosis and apoptosis. Based on H9C2 cell studies, they suggested that the protective effect occurred via regulation of the SIRT1/Parkin/mitophagy pathway. These findings, however, contradict the earlier finding by Kyhl *et al*. (2017) that a similar liraglutide treatment in rats had no effect on adverse remodeling or cardiac function^26^. Given the need to develop new methods for mitigating adverse post-MI remodeling, the existing controversy regarding the effectiveness of GLP1R agonists in limiting this process and the absence of information regarding their mechanism of action in the setting of intact animals, the purpose of this study was to investigate whether brief intermittent administration post-MI of the GLP1R agonist DMB could induce mitochondrial turnover and mitigate post-MI adverse remodeling.

## Results

### Post-infarction administration of DMB mitigates adverse remodeling and improves cardiac function

**Figure 1:**
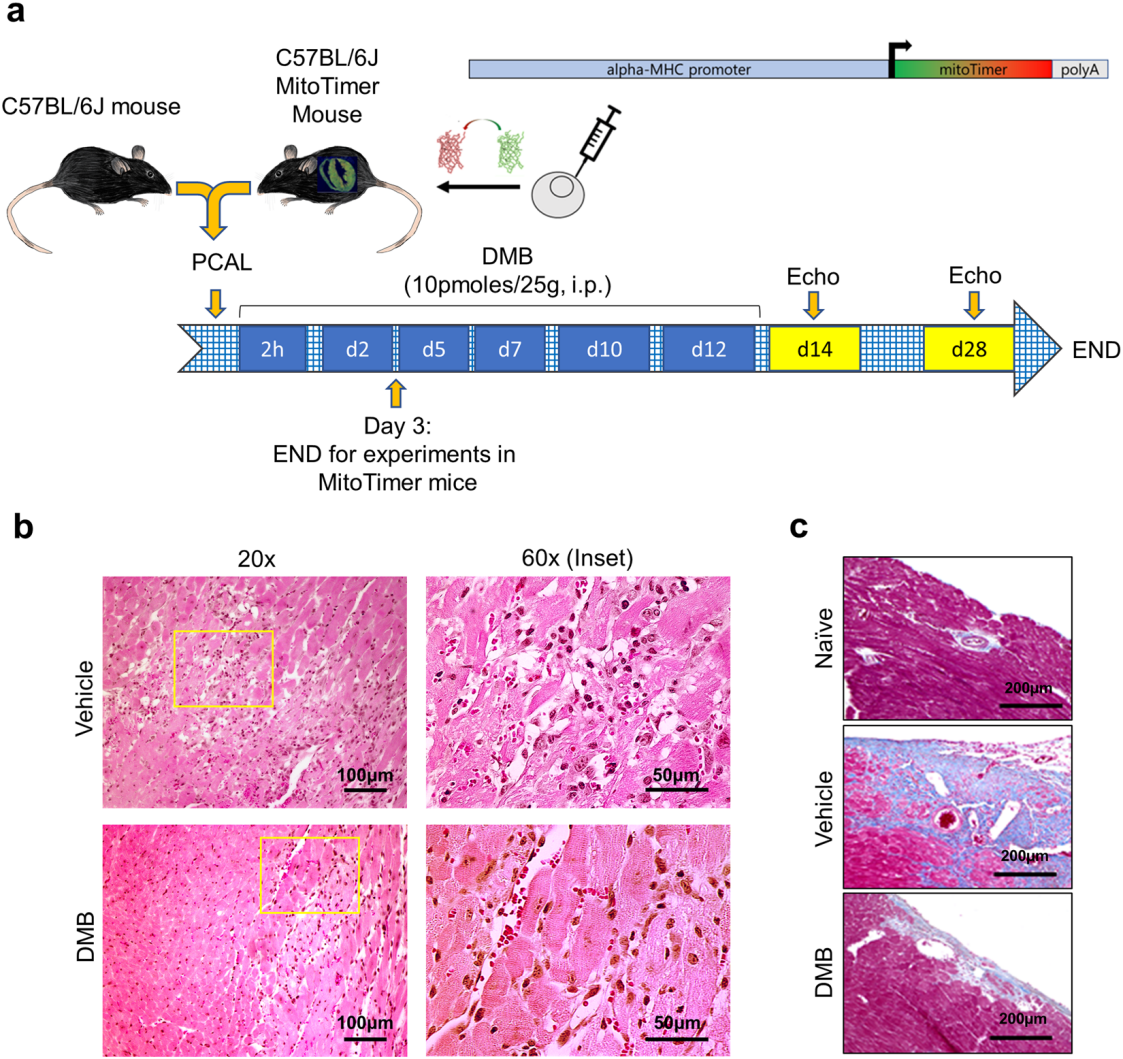
DMB attenuates immune cell infiltration and fibrosis. Age-matched wild-type (WT) and MitoTimer mice underwent permanent coronary artery ligation (PCAL) and 2 hours later were given vehicle (50 µL DMSO) or DMB (10 pmoles/25 g) i.p. three times a week for two weeks (total 6 doses). **(a)** Schematic figure of the protocol; **(b)** Representative 60X and 20X magnification of heart sections stained with H&E 3 days after PCAL at the border zone of WT mouse hearts to highlight infiltration of immune cells. (n = 4 animals/group). **(c)** Representative 20X magnification of WT heart sections stained with Masson Trichrome showing fibrosis 7 days after PCAL. Scale bars as indicated.

A schematic of the experimental procedure detailing our mouse models of permanent coronary artery ligation and DMB treatment scheme is shown in Fig. 1a. Immune cell infiltration 3 days post-permanent coronary artery ligation was less in the cohort of mice treated with DMB (Fig. 1b). Staining of hearts with Masson-Trichrome at 7 days post-permanent coronary artery ligation revealed a marked reduction in interstitial fibrosis in DMB-treated mice (Fig. 1c). Echocardiographic studies performed 14 and 28 days post-MI showed significant improvements in EF%, FS%, LVPW,d and LVPW,s (Table 1), although improvement did not reach baseline values from naïve mice (EF ~60% and FS ~30%, see Table 1 legend). Overall, the hearts of control mice noticeably exhibited ventricular wall thinning and were enlarged relative to vehicle controls, albeit there were no differences noted in body weights, heart weights and lung wet-to-dry weight ratios of mice treated with DMB or vehicle (Supplementary Figure S1) 14 and 28 days after procedure. At the doses used, DMB did not cause hypoglycemia (Supplementary Figure S1).

**Table 1:** DMB administered after permanent coronary artery ligation mitigates adverse LV remodeling and improves cardiac function.

		14 days post-MI		28 days post-MI	
		Vehicle n = 6	DMB n = 7	Vehicle n = 6	DMB n = 7
HR	bpm	432 ± 32.2	467 ± 50.1	446 ± 22.7	478 ± 37.3
EF	%	39.6 ± 4.5	49.0 ± 5.9*	32.9 ± 5.5	42.3 ± 5.3*
FS	%	19.3 ± 2.4	24.7 ± 3.5*	15.6 ± 2.8	21.0 ± 3.1*
LV Vol,d	uL	113.4 ± 39.7	96.9 ± 23.3	110.4 ± 38.5	125.0 ± 22.1
LV Vol,s	uL	69.3 ± 27.4	49.9 ± 14.0	75.4 ± 31.3	71.8 ± 12.8
LVID,d	mm	4.85 ± 0.73	4.56 ± 0.51	4.8 ± 0.7	5.1 ± 0.4
LVID,s	mm	3.93 ± 0.66	3.44 ± 0.46	4.1 ± 0.7	4.0 ± 0.3
LVPW,d	mm	1.04 ± 0.26	1.25 ± 0.31	1.2 ± 0.3	1.7 ± 0.3*
LVPW,s	mm	1.21 ± 0.24	1.48 ± 0.31	1.6 ± 0.4	2.1 ± 0.2*
Est. scar size	%	34.3 ± 3.0	28.8 ± 7.1	34.5 ± 6.8	30.3 ± 7.1

Data are presented as mean ± standard deviation. HR, Heart rate; EF, Ejection Fraction; FS, Fractional Shortening; DMB, 2-Quinoxalinamine, 6,7-dichloro-N-(1,1-dimethylethyl)-3-(methylsulfonyl)-, 6,7-dichloro-2-methylsulfonyl-3-N-tert-butylaminoquinoxaline; LV Vol,d, Left Ventricle End-Diastolic Volume; LV Vol,s, LV End- Systolic Volume; LVID,d, LV Internal Diameter, Diastole; LVID,s, LV Internal Diameter, systole; LVPW,d, LV Posterior Wall thickness, diastole; LVPW,s, LV Posterior Wall thickness, systole. Naïve EF % 60.4 ± 6.7, FS % 29.3 ± 6.3 (n = 19). *p < 0.05 vs vehicle.

### DMB enhances cardiac autophagy and mitophagy flux

**Figure 2:**
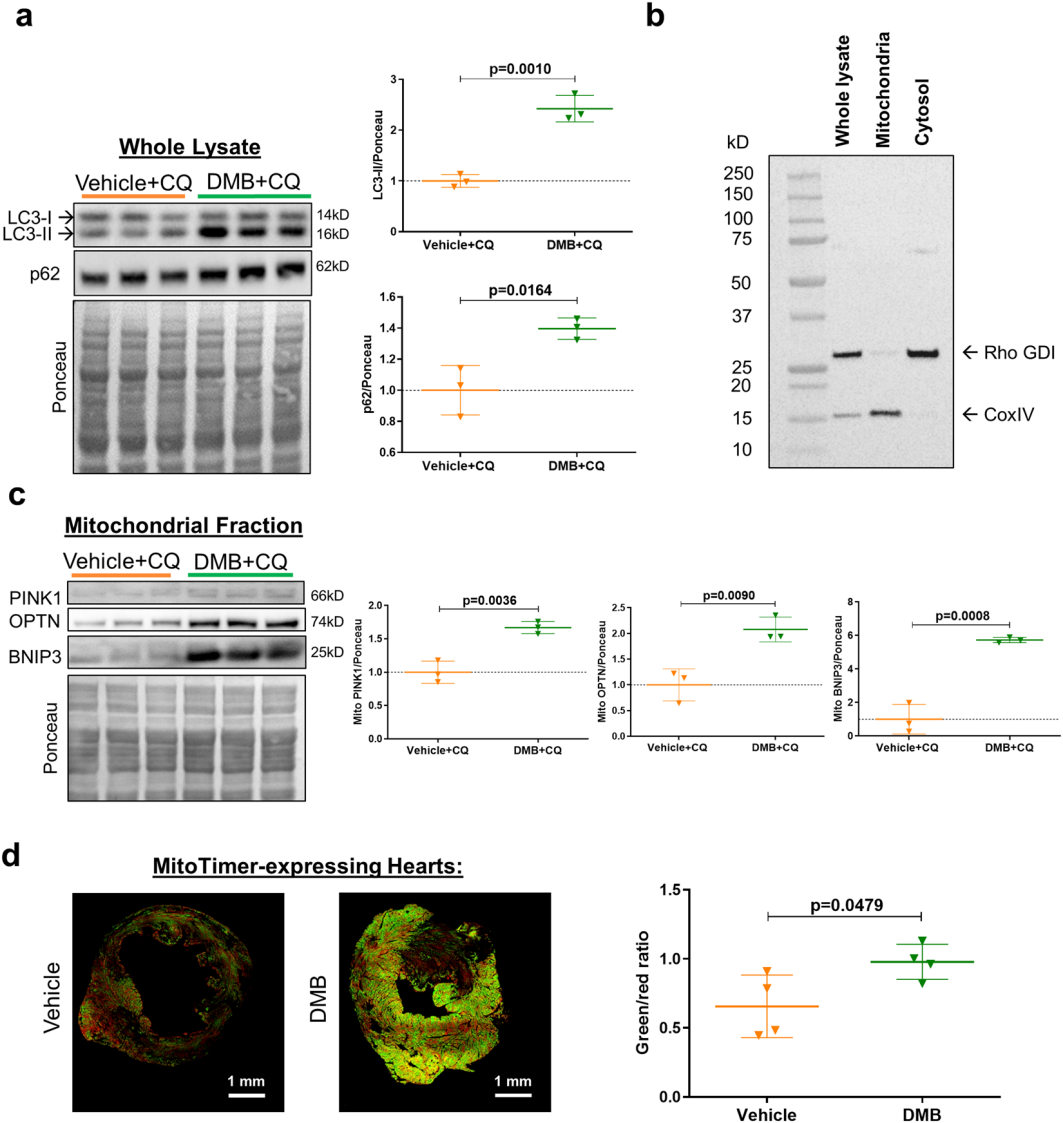
DMB induces cardiac autophagy and mitophagy *in vivo*. Wild type (WT) mice received vehicle (50 µL DMSO) or DMB (10 pmoles/25 g) i.p. One hour later, mice received chloroquine (CQ, 10 mg/kg i.p.) and were sacrificed 16 hours later (n = 3/group). Hearts were harvested and cellular fractions were collected. **(a)** Western blot analysis and quantification of autophagy markers LC3-II and p62 in heart whole lysates; **(b)** Western blot of subcellular fractions showing the purity of the mitochondria-enriched fraction. **(c)** Western blot analysis and quantification of mitophagy markers PINK1, Optineurin (OPTN) and BNIP3 in the mitochondrial fraction; **(d)** Age-matched WT MitoTimer mice were subjected to permanent coronary artery ligation and given DMB (10 pmoles/25 g) or vehicle (DMSO 50 µL) i.p. 2 hours after surgery and once more 2 days later. Animals were sacrificed 3 days after PCAL. Cryopreserved sections of the heart were examined for MitoTimer ratiometric shift (green/red ratio = new/old mitochondria) as an indication of mitochondrial biogenesis (n = 4 mice/group). Scale bars as indicated. All values are presented as mean ± standard deviation. Standard Student’s *t*-test was used to compare the groups. WB figures were cropped and all densitometry was performed using NIH Image J software v 1.51 (https://imagej.nih.gov/ij/download.html). Full-length blots/gels are presented in Supplementary Figure 3.

To investigate if DMB stimulates cardiac autophagy, we treated naïve wild-type mice with the drug or its vehicle (DMSO), followed by chloroquine to prevent lysosomal degradation of cargo. As seen in Fig. 2a, DMB treatment increased p62/SQSTM1 and LC3-II in post-nuclear supernatants (whole lysates), suggesting increased autophagy. We obtained mitochondria-enriched heavy membrane fractions (Fig. 2b) to examine accumulation of proteins known to facilitate mitophagy. Biochemical evidence of mitophagy was indicated by increased PINK1, optineurin, and Bnip3 in the mitochondria-enriched heavy membrane fractions (Fig. 2c). MitoTimer mice subjected to permanent coronary artery ligation and treated with DMB exhibited a marked increase in MitoTimer fluorescence and an increased green:red ratio when compared to vehicle-treated controls 3 days post-MI (Fig. 2d). These findings highlight the ability of DMB to enhance mitochondrial turnover in the setting of ischemic injury.

### DMB directly stimulates autophagic and mitophagic flux in differentiated H9C2 cardiomyocytes

**Figure 3:**
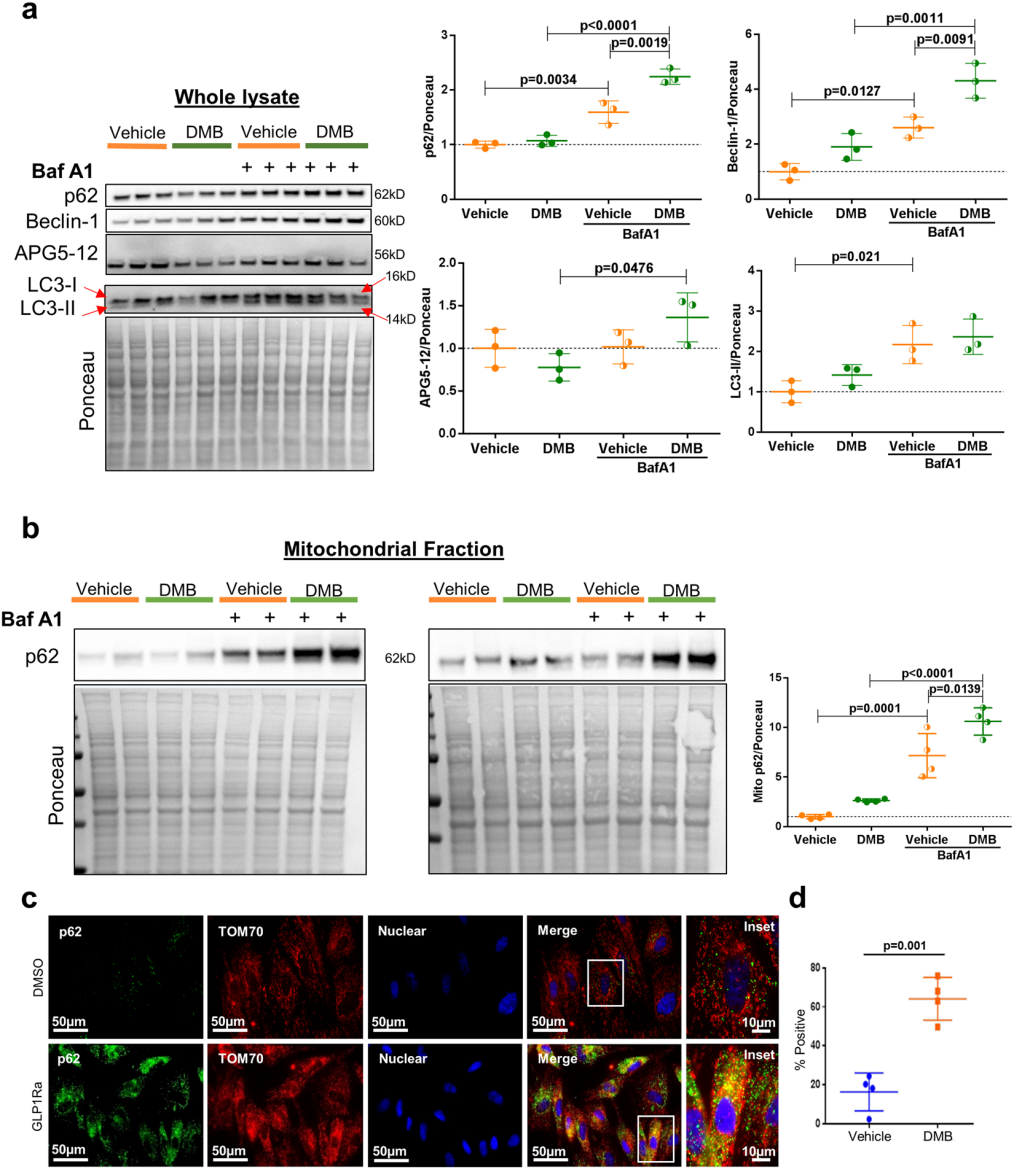
DMB induces mitophagy in H9C2 cells. Differentiated H9C2 cells were treated with vehicle (DMSO) or 1 µM DMB overnight with or without autophagy inhibitor bafilomycin A1 (BafA1, 50 nM). **(a)** Western blot analysis and quantification of autophagy markers in the cell whole lysate (n = 3 plates/group); **(b)** Western blot analysis and quantification of p62 in the mitochondria-enriched subcellular fraction of DMSO or DMB-treated cells with or without BafA1 (n = 4 plates/group). The samples derive from the same experiment. The blots were processed in parallel and developed using the same exposure time. **(c)** Immunofluorescence microscopy of the mitophagy adapter p62/SQSTM1 and the mitochondrial protein TOM70 in DMSO and DMB-treated H9C2 cells, scale bars as indicated; **(d)** Percentage of cells showing colocalization of p62 and TOM70. (n = 4 plates/group with 10 fields of cells captured for each plate. Scoring was performed blinded. All values are presented as mean ± standard deviation. ANOVA with Tukey posthoc test was used to compare the groups in A and B. Standard Student’s *t*-test was used to compare the groups in D. WB figures were cropped and all densitometry was performed using NIH ImageJ software v 1.51 (https://imagej.nih.gov/ij/download.html). Full-length blots/gels are presented in Supplementary Figure 3.

To assess autophagy and mitophagy, we treated H9C2 cells with DMB or vehicle and lysosomal blockade. Western blots of lysates from DMB-treated cells revealed increased autophagic flux indicated by accumulation of p62/SQSTM1 and Beclin-1 in the presence of bafilomycin, although APG5-12 complex and LC3-II accumulation were not different (Fig. 3a). Western blot analysis of mitochondria-enriched heavy membrane fractions showed translocation of mitophagy adapter p62/SQSTM1 after lysosomal blockade, which was enhanced by DMB (Fig. 3b). Moreover, immunofluorescence microscopy verified that DMB increased the colocalization of p62/SQSTM1 with mitochondria (Fig. 3c,d). DMB lowered mitochondrial membrane potential and induced mitochondrial fragmentation; conditions that precede and facilitate mitophagy (Supplementary Figure S2)^27–29^. These results correlate with our findings *in vivo* and demonstrate that DMB can directly induce autophagy and mitophagy in cardiac cells in a cell-autonomous manner.

### Beneficial effects of DMB are lost in parkin knockout mice

**Figure 4:**
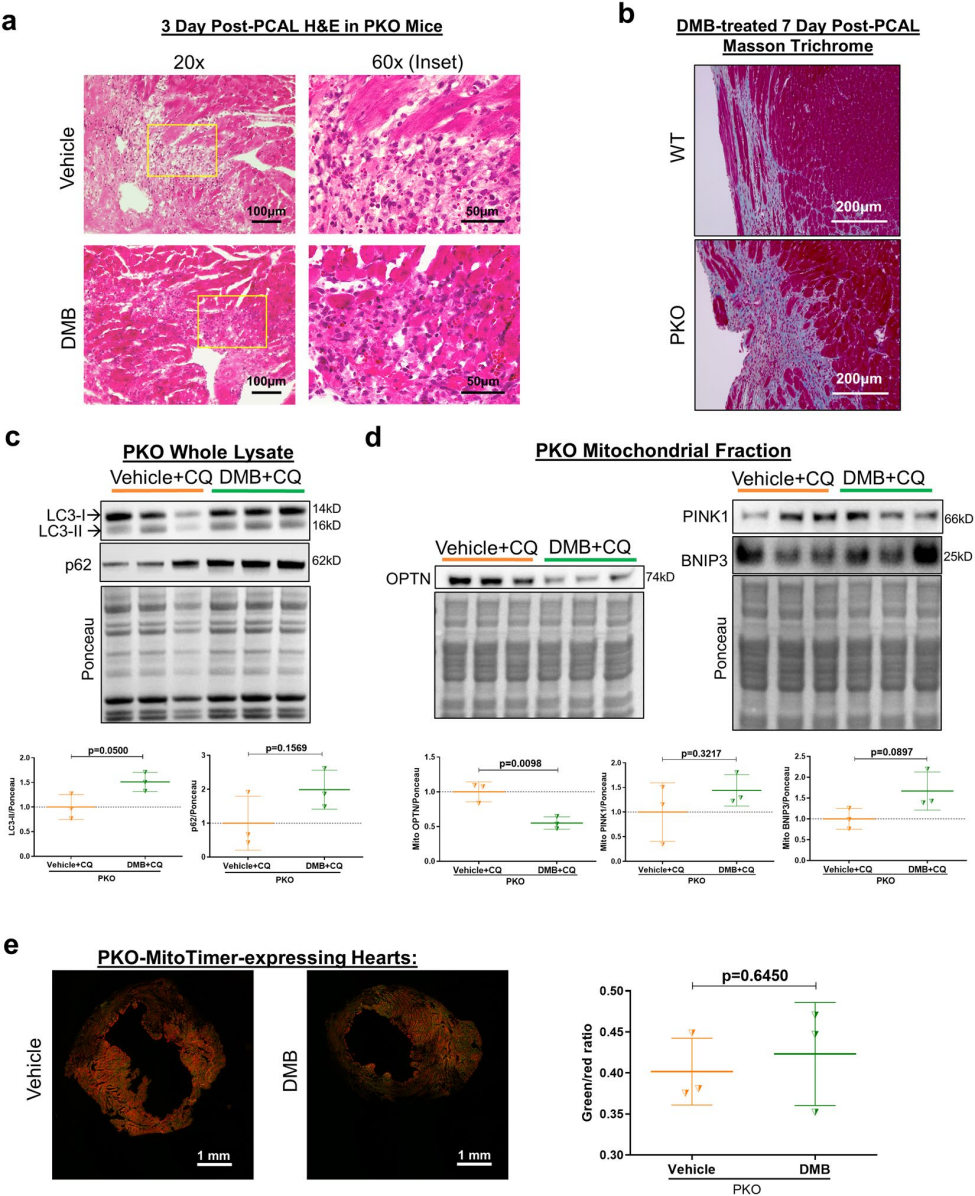
DMB fails to limit inflammation, fibrosis and induce mitochondrial turnover in PKO mice. Age-matched PKO mice underwent permanent coronary artery ligation and were given vehicle (50 µL DMSO) or DMB (10 pmoles/25 g) i.p. 2 hours after surgery and again 2 and 5 days later. Mice were sacrificed 3 days after PCAL (n = 3–4 mice/group) for H&E staining and MitoTimer ratiometric shifts, and 7 days after PCAL for Masson Trichrome staining. A second cohort of mice received vehicle (50 µL DMSO) or DMB (10 pmoles/25 g) i.p. One hour later, mice received chloroquine (CQ, 10 mg/kg i.p.) and were sacrificed 16 hours later (n = 3/group) for western blot analysis of autophagy and mitophagy markers. **(a)** Representative 60X and 20X magnification of heart sections stained with H&E showing immune cell infiltration in PKO mice 3 days post-MI; **(b)** Representative 20X magnification of heart sections with Masson Trichrome 7 days after PCAL showing fibrosis in PKO *vs*. wild type in DMB-treated mice. **(c)** Western blot analysis and quantification of autophagy markers p62 and LC3 in heart whole lysates of PKO mice; **(d)** Western blot analysis and quantification of mitophagy markers PINK1, Optineurin (OPTN) and BNIP3 in the mitochondrial-enriched fraction of PKO mice; **(e)** Cryopreserved sections of the heart were examined for MitoTimer ratiometric shifts (green/red ratio = new/old mitochondria) as an indication of mitochondrial biogenesis (n = 4 mice/group). All values are presented as mean ± standard deviation. Scale bars as indicated. Standard Student’s *t*-test was used to compare the groups. WB figures were cropped and all densitometry was performed using NIH Image J software v 1.51 (https://imagej.nih.gov/ij/download.html). Full-length blots/gels are presented in Supplementary Figure 3.

**Figure 5:**
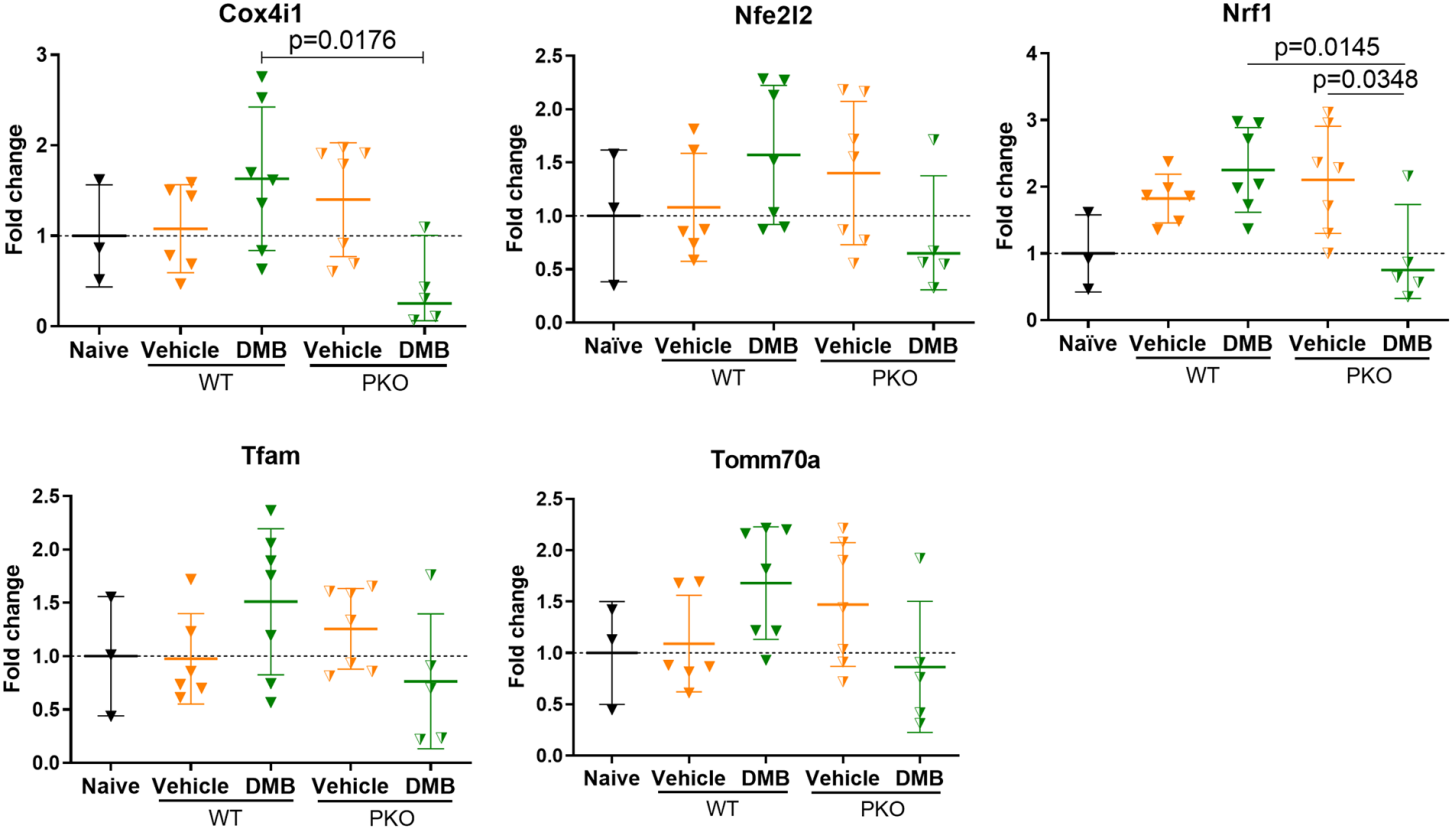
PKO mice exhibit decreased DMB-associated mitochondrial biogenesis gene expression. Age-matched wild-type and PKO mice underwent permanent coronary artery ligation and were given vehicle (50 µL DMSO) or DMB (10 pmoles/25 g) i.p. 2 hours after surgery and again 2 days later. Mice were sacrificed 3 days after PCAL (n = 3–7 mice/group) for gene expression analysis. All values are presented as mean ± standard deviation. ANOVA with Tukey posthoc test was used to compare the groups. Gene expression was normalized to the average expression of 18 S and Rpl4.

Unlike wild-type mice, GLP1Ra treatment in Parkin knockout (PKO) mice failed to attenuate immune cell infiltration into the border zone (Fig. 4a) or limit fibrosis (Fig. 4b). Subsequently, wild-type and PKO mice were studied in separate cohorts to assess potential beneficial effects of DMB on contractility; wild-type were not directly compared with PKO mice. As shown in Table 2, treatment had no effect on post-infarction remodeling (LVPW,d or LVPW,s) or cardiac function as measured out to 14 days post-PCAL procedure. We limited studies in PKO mice to 14 days post-MI due to high morbidity and mortality in the vehicle treated group: in one cohort, 3 of 10 PKO mice died from LV rupture within 14 days, compared to only 1 of 10 wild-type mice. The increased susceptibility of PKO mice to succumb to cardiac ischemic challenge is in line with prior findings^30^. Naïve PKO mice given DMB (and subsequently chloroquine to limit autophagy/mitophagic flux) accumulated p62/SQSTM1 but not LC3-II in whole lysates (Fig. 4c). Compared to wild-type mice, DMB treatment in PKO mice also failed to trigger increased PINK1, Optineurin or Bnip3 translocation to the mitochondria (Fig. 4d). Finally, DMB treatment of PKO-MitoTimer mice did not shift their green:red ratio, reflecting impaired mitochondrial turnover (Fig. 4e). To further investigate if mitochondrial biogenesis was differentially affected between wild-type and PKO mice, we compared mRNA expression of targets downstream of PGC-1α on day 3 after permanent coronary artery ligation. We found that there was a trend for DMB to increase mRNA levels of Cox4i1, Nfe2l2, Nrf1, Tfam and Tomm70a. Conversely, these targets either trended to be decreased or were significantly decreased by DMB treatment in PKO mice (Fig. 5).

**Table 2:** DMB administered after permanent coronary artery ligation fails to mitigate MI-induced adverse LV remodeling and improve cardiac function in PKO mice.

		Wild-type		PKO	
		Vehicle n = 4	DMB n = 4	Vehicle n = 4	DMB n = 4
HR	bpm	410 ± 58.9	481 ± 65.1	431 ± 64.9	492 ± 55.6
EF	%	36.8 ± 2.7	45.2 ± 4.5*	30.5 ± 7.8	32.4 ± 7.2
FS	%	17.6 ± 1.4	22.5 ± 2.5*	14.5 ± 3.7	15.5 ± 3.6
LV Vol,d	uL	99.7 ± 23.2	107.5 ± 34.1	129.7 ± 62.3	145.7 ± 55.0
LV Vol,s	uL	63.2 ± 15.7	59.7 ± 23.4	92.4 ± 52.8	101.0 ± 49.5
LVID,d	mm	4.6 ± 0.5	4.8 ± 0.6	5.1 ± 1.0	5.4 ± 0.9
LVID,s	mm	3.8 ± 0.4	3.7 ± 0.6	4.4 ± 1.0	4.6 ± 0.9
LVPW,d	mm	0.8 ± 0.1	0.9 ± 0.1	0.7 ± 0.2	0.8 ± 0.1
LVPW,s	mm	1.0 ± 0.1	1.2 ± 0.2	1.0 ± 0.2	1.0 ± 0.2
Est. scar size	%	35.5 ± 5.1	32.4 ± 6.9	38.4 ± 6.4	31.2 ± 2.7

Echocardiograms were analyzed on day 14 post-permanent coronary artery ligation. Data is presented as mean ± standard deviation. HR, Heart rate; EF, Ejection Fraction; FS, Fractional Shortening; DMB, 2-Quinoxalinamine, 6,7-dichloro-N-(1,1-dimethylethyl)-3-(methylsulfonyl)-, 6,7-dichloro-2-methylsulfonyl-3-N-tert-butylaminoquinoxaline; LV Vol,d, Left Ventricle End-Diastolic Volume; LV Vol,s, LV End- Systolic Volume; LVID,d, LV Internal Diameter, Diastole; LVID,s, LV Internal Diameter, systole; LVPW,d, LV Posterior Wall thickness, diastole; LVPW,s, LV Posterior Wall thickness, systole. Naïve EF % 60.4 ± 6.7, FS % 29.3 ± 6.3 (n = 19); PKO baseline - EF % 56.2 ± 4.5, FS % 27.8 ± 2.3 (n = 9). *p < 0.05 vs vehicle.

## Discussion

The salient findings in this study are that (1) short-term intermittent administration of the quinoxaline GLP-1 receptor agonist (DMB) after infarction attenuates adverse remodeling and improves cardiac function; (2) DMB enhances autophagy, mitophagy, and mitochondrial biogenesis; (3) the salutary effects of DMB on remodeling were lost in Parkin knockout mice, suggesting that the beneficial effects are dependent on Parkin-mediated mitophagy and (4) Parkin loss also negatively impacts DMB-mediated mitochondrial biogenesis. Because a permanent ligation model was used, and treatment was instituted after infarction, treatment was unlikely to modify infarct size, indicating that DMB directly modulates the remodeling process. The major strengths of this study include (i) short-course intermittent administration of an allosteric quinoxaline GLP-1 receptor agonist; (ii) use of an *in vivo* genetic model with impaired mitophagy (Parkin KO mice); (iii) use of Mito-Timer mice and Mito-Timer Parkin KO mice; (iv) analysis of specific markers of mitophagy including optineurin, Bnip3 and Pink1; and (v) use of bafilomycin and chloroquine to assess mitophagic flux in cells and intact mice, respectively.

The allosteric quinoxaline GLP-1 receptor agonist DMB (C_13_H_15_Cl_2_N_3_O_2_S; Molecular Weight 348.25 gm/mol) is distinctly different from the long acting GLP-1 peptide analogs exenatide (C_184_H_282_N_50_O_60_S; Molecular Weight 3,751.62 g/mol) and liraglutide (C_172_H_265_N_43_O_51_; Molecular Weight 4,186.63 g/mol). Because we administered DMB only 3×/week, we assume that we have stimulated episodic bouts of mitophagy with time to replace mitochondria through biogenesis. The intermittent nature of our intervention contrasts with and may explain the findings by Kyhl *et al*. (2017) that a 4-week course of the long-acting peptide analog liraglutide post-MI in rats failed to mitigate adverse remodeling. Moreover, there are reports that use of the current marketable forms of longer half-life peptide GLP-1 receptor agonists are associated with complications such as the risk of hypoglycemia, abdominal pain, pancreatitis, pancreatic cancer and thyroid cancer^18,19^. Our use of a small molecule quinoxaline GLP1Ra with a shorter half-life, intermittent dosing, and for a duration of only 2 weeks would minimize the risk of inducing such complications.

The concept that autophagy and mitophagy play important roles in preventing adverse remodeling is credible given that these homeostatic processes are known to limit the severity of ischemic injury^30–34^. Buss *et al*. (2009) found that rats treated with everolimus (an mTOR inhibitor and autophagy inducer) after permanent coronary artery ligation stimulated cardiac autophagy and this was associated with less adverse remodeling^35^. Kanamori *et al*. (2011) found enhanced autophagic activity in the border zone one week after permanent coronary artery ligation^36^ and that bafilomycin A1 (an autophagy inhibitor) exacerbated post-MI cardiac dysfunction and remodeling, while rapamycin (an autophagy enhancer) had the opposite effect. Wu *et al*. (2014) also found an increase in autophagy during the acute MI phase but noted a decline beginning 5 days after ligation. Rapamycin attenuated, while 3-methyladenine (an autophagy inhibitor) exacerbated, post-MI remodeling and cardiac dysfunction. They concluded autophagy plays a role in mitigating adverse remodeling, but its effectiveness was impaired post-MI^37^. A key concept illustrated in their work is that transient upregulation of autophagy leads to changes in the mitochondrial population which is associated with improved energetics and cardiac function. Thus, there is ample evidence to implicate targeting mitochondrial turnover as a viable means for attenuating the development of post-MI remodeling.

We also sought to investigate possible cellular pathways impacted by Parkin loss that could help explain the failure of DMB to attenuate adverse remodeling in PKO mice. Western analysis of heart lysates from 3-day post MI mice revealed that cellular energetics seemed largely unaffected as activation of AMPK (phospho-AMPK, Thr 172) was equal in both wild-type and PKO mice and unchanged with DMB treatment. Likewise, activation of mTOR (phospho-mTOR Ser2448) was comparable between wild-type and PKO mice (data not shown). However, loss of Parkin is known to limit tolerance of the heart to ischemic or oxidative challenge due to impaired mitophagy^30,31,38^. More recently, the concept has emerged that loss of Parkin also negatively impacts PGC-1α-related mitochondrial biogenesis due to lost degradation of PARIS or that it directly is involved in mitochondrial DNA replication^39–41^. In line with these reports, we also noticed that transcriptional targets related to mitochondrial biogenesis were diminished in response to DMB treatment in PKO mice. Our work here supports these concepts and shows that Parkin affects the two main components of mitochondrial turnover: mitophagy and biogenesis. Moreover, this suggests that there exists a relationship between mitochondrial quality control and the development of post-MI adverse LV remodeling.

Our study demonstrates the importance of Parkin-mediated mitophagy in the mechanism of DMB-mediated mitigation of adverse remodeling. However, several limitations of the study should be noted. While the fluorescence microscopy and biochemical data in our *in vitro* studies provide evidence that DMB induces mitophagy in cardiac cells, these methods are indirect relative to assays utilizing mitochondria-targeted Keima or RFP-GFP constructs. Moreover, Parkin deficiency may impact other cellular processes in addition to mitophagy that are part of the mechanism of DMB action. Reliance on global Parkin knockout mice in this study also may not account for the effects of Parkin loss beyond the heart nor for compensatory mechanisms developed by the strain. It is possible that the benefits of post-MI DMB may go beyond the cardiomyocyte and involve macrophages, fibroblasts, endothelial cells, the innate immune response, or other tissues. Thus, identifying other cellular programs affected by DMB that affect the remodeling process is paramount for defining and prioritizing target pathways to modulate. While some studies have examined the pharmacological properties of DMB in comparison to existing GLP1R agonists^20–22,42,43^, much work remains to characterize the utility and side effects (positive or negative) of using DMB. Such studies may include but are not limited to examining half-life of the drug in circulation, its effects on ROS, ER stress, cardiac cell tolerance to hypoxia and ischemia-reperfusion induced cell death, performing more detailed head-to-head comparisons with existing GLP1R agonists, and noting potential harmful effects, if any. Of interest to our group is elucidating the mechanism by which DMB elicits autophagy and mitochondrial turnover.

In summary, despite the fact that adverse post-MI left ventricular (LV) remodeling is the major cause of HF in the US, very little progress has been made in the prevention or treatment of this process. Here we report that short-term intermittent administration of DMB given post-MI is effective in mitigating adverse remodeling via a mechanism that is dependent on Parkin. This approach is clinically applicable and helps avoid the potential adverse effects that may arise from chronic GLP-1 receptor agonism.

## Materials and Methods

### Animal ethics

Animal procedures followed the National Institutes of Health – Guide for the Care and Use of Laboratory Animals (8^th^ edition) and were approved by the Institutional Animal Care and Use Committee of Cedars-Sinai Medical Center. C57BL/6J male mice between 12–13 weeks old were used. Mice exhibiting discomfort and distress were treated with buprenorphine (1 mg/kg, i.p.) or euthanized when appropriate. At the end of experimental procedures, mice were anesthetized via isoflurane and euthanized by cervical dislocation. Depth of anesthesia was confirmed by loss of flexor muscle response from paw pinch.

### Mouse protocols

#### Permanent coronary artery ligation and DMB treatment

A mouse model was used to assess the effect of DMB administration after permanent coronary artery ligation on the development of adverse LV remodeling (Fig. 1a). Briefly, C57BL/6J wild-type and PKO mice (same background) were anesthetized with isoflurane (1%), ketamine (50 mg/kg) and xylazine (10 mg/kg), intubated and ventilated. Pressure-controlled ventilation (Harvard Apparatus) was maintained at 2–3 cm H_2_O throughout the procedure. Anesthesia was maintained with 0.5–1% isoflurane. A left thoracotomy was performed to expose the heart. The pericardium was opened, and a 7-0 silk suture was placed around the proximal left coronary artery 2 mm below the left atrial appendage using a curved 27.5 G needle. The vessel was ligated, chest closed, and animals allowed to recover. Two hours later, mice were randomized to receive intraperitoneal (i.p.) injections of 50 μl of vehicle (DMSO) or 10 pmoles/25 g mouse of DMB (Sigma, G8048). The surgeon performing the procedure and performing echo assessments was blinded to treatment condition. This dose we chose to examine here was based on pilot data aimed to deliver a dose that reliably induced cardiac mitophagy and was tolerated in mice that had undergone PCAL. However, we found that doses of up to 720 pmole per bolus was also effective at mitigating adverse remodeling (data not shown) but chose to stay with the lowest dose tested that conferred benefit (10 pmoles). The dose chosen (10 pmoles/dose) did not affect glucose levels (Supplementary Figure S1). Injections were repeated 3 times a week for a total of 6 injections. Body weights were recorded prior to permanent coronary artery ligation and afterwards on days 3, 7, 14 and 28. Following echo assessment, mice were sacrificed 14 and 28 days after MI. Lungs and hearts were harvested and weighed to assess wet/dry ratio (lungs); and heart weights were normalized to tibia length. For biochemistry, the largely acellular fibrous infarct region was excluded. In separate experimental cohorts, hearts were harvested 3 and 7 days post-MI for histological assessment of immune cell infiltration and fibrosis, respectively. We limited studies in PKO mice to 14 days post-MI due to high morbidity and mortality: in one cohort, 3 of 10 PKO mice died from LV rupture within 14 days, compared to only 1 of 10 wild-type mice.

#### DMB effects on cardiac autophagy/mitophagy

To determine the effect of DMB on cardiac autophagy and mitophagy, naïve mice were given a single dose of DMB or vehicle, followed one hour later with chloroquine (10 mg/kg in saline, i.p.). Hearts were harvested the next day (~16 hours later) and processed for western blot analysis.

#### MitoTimer mice and mitochondrial turnover

Previously, we showed that MitoTimer mice are useful for monitoring stimulation of mitochondrial turnover^44–46^. Age-matched wild-type MitoTimer mice were subjected to PCAL and given DMB (10 pmoles/25 g) or vehicle (DMSO 50 µL) i.p. 2 hours after surgery and once more 2 days later. Animals were sacrificed 3 days after PCAL. Hearts were excised and quickly rinsed with PBS to wash out excess blood then fixed in 4% formalin overnight, embedded in OCT compound and then allowed to freeze at −80 °C for at least 24 hours. Cryopreserved sections of the heart were then prepared. Sections within the border zone region were examined for MitoTimer ratiometric shift (green/red ratio = new/old mitochondria) as an indication of mitochondrial biogenesis as previously described^46^. Cryo-embedded heart tissue from 3 day post-permanent coronary artery ligation and DMB treatment in wild-type or PKO MitoTimer mice were examined for ratiometric changes in green:red fluorescence.

#### Echocardiography

Transthoracic echocardiography was performed with a high-resolution Vevo 3100 (Fujifilm VisualSonics, Inc.). Mice were anesthetized with an inhaled mixture of oxygen and 2% isoflurane gas at a flow rate of 1 L/min. The short-axis two-dimensional view of the heart was obtained at the level of the papillary muscles. M-mode images were recorded. This method of analyzing cardiac function has been validated by others^47–51^. Heart rate was determined from cardiac cycles: typically, 400 to 500 bpm. Ultrasound gel was warmed to body temperature before application to the depilated mouse chest. All LV dimensions are presented as the average of measurements of 3 consecutive sinus beats using the leading-edge technique. Ejection Fraction (EF)% was then calculated from M-mode-derived LV dimensions using the formula (LV Volume = [7/(2.4 + LVID)] * LVID3 EF = (LV vol,d-LV vol,s)/LV vol,d *100. Fractional Shortening (FS) = [(LVd-LVs)/LVd)]*100.

### Histology

On days 3 and 7 after permanent coronary artery ligation, hearts were harvested, perfused with PBS and stored in 4% paraformaldehyde (PFA) for 24 hours, followed by 70% ethanol storage and cryo embedding in optimal cutting temperature compound for subsequent histopathology staining and microscopic analysis (Keyence Biorevo BZ-9000). To identify immune cell infiltration on day 3, slide sections were stained using the Hematoxylin and Eosin Stain Kit (Vector Laboratories Inc.) according to manufacturer’s protocol, followed by dehydration in 95–100% ethanol gradient. Slides were cleared with xylene and mounted with Cytoseal. To visualize fibrosis on day 7 post-MI, sections were stained following manufacturer’s protocol for Masson’s Trichrome (Sigma). Briefly, slide sections were deparaffinized using xylene and an ethanol gradient (100–70%), followed by hydration in deionized water. Sections were placed in a container with Bouin solution overnight, washed and stained in Weigert’s iron hematoxylin and Biebrich scarlet-acid fuchsin. Further steps included decolorizing with phosphomolybdic/phosphotungstic acid solution, aniline blue staining and clarification in 1% acetic acid solution. Sections were dehydrated in ethanol 95–100%, cleared in xylene and mounted.

### Subcellular fractionation for mitochondrial enrichment

Mitochondrial fractionation from heart tissue and differentiated H9C2 cells followed the protocol previously described by Andres and colleagues^52^. For harvesting H9C2 cells, media was removed, and then cells were rinsed once with PBS. HES buffer with appropriate inhibitors was then added and cells scraped off and transferred to an Eppendorf tube. Cells were lysed using a 27.5 G syringe needle, then followed mitochondrial fractionation. Prepared samples were stored at −80 **°**C until use. Our group has used this method to obtain mitochondria-enriched heavy membrane fractions^31,45^.

### Western blot analysis

Protein concentrations of lysates were determined by Bio-Rad DC Protein Assay kit. Equal amounts of proteins were resolved on Bolt 4–12% Bis-Tris Plus gels (Thermo Fisher Scientific) and transferred to nitrocellulose membranes. Membranes were blocked with 5% nonfat dry milk for 1 hour, then incubated with 1:1000 diluted primary antibodies against: Optineurin and APG5-12 conjugate (ATG5, Santa Cruz Biotechnology), LC3, Beclin-1, Bnip3, PINK1, and p62/SQSTM1 (Abcam) at 4 **°**C overnight. Membranes were washed with Tris-buffered saline pH 7.6 containing Tris HCl (20 mM) and NaCl (150 mM) with 0.1% Tween-20 (Sigma-Aldrich) (TBS-T) at room temperature and incubated with KPL Peroxidase Labeled Goat anti-Mouse IgG (H + L) (1:3000, KPL Affinity Purified Antibody, SeraCare) or KPL Peroxidase Labeled Goat anti-Rabbit IgG (H + L) secondary antibodies for 2 hours. Membranes were rinsed in TBS-T (3 × 5 min). Blots were developed with Clarity Western ECL Substrate (Bio-Rad) and imaged using a ChemiDoc XRS and the Image Lab software v 5.0 (Bio-Rad). Densitometry was performed using NIH Image J software v 1.51 (https://imagej.nih.gov/ij/download.html).

### H9C2 rat ventricular cardiomyocytes

H9C2 cells were obtained from ATCC and maintained in growth media (DMEM: 10 mM glucose, 10% FBS, antibiotic and antimycotic, pH 7.4). Once cells were ~90% confluent, differentiation was initiated by switching to differentiation media (DMEM: 10 mM glucose, 1% FBS, antibiotic and antimycotic, 1 nM retinoic acid, pH 7.4) in the manner described by Jaishy *et al*. 2015^53^. Differentiation was sustained for 5 days before starting experiments. To investigate if DMB induces autophagy and/or mitophagy, 1 µM DMB was added to the medium for 24 hours. 8 hours before cell harvest, 50 nM Bafilomycin A1 or vehicle (DMSO 0.1% vol) was added to inhibit lysosomal degradation for flux determination.

### Immunofluorescence and microscopy

H9C2 cells were seeded on fibronectin-gelatin coated glass bottom microwell dishes (MatTek) at a density of 2.5 × 10^3^ per well and differentiated for five days. Cells were then pretreated with 1 μM DMB or vehicle (DMSO) for 16 hours and then with 1 μM DMB or vehicle (DMSO) with 50 nM Bafilomycin A1 for additional 8 hours. Cells were fixed with 4% formaldehyde (Sigma-Aldrich) in PBS for 10–15 min at room temperature then washed with PBS. Nuclei were stained with Hoechst 33342 (Invitrogen) and rinsed with PBS. Cells were permeabilized with PBS containing 5% horse serum, 5% goat serum and 0.3% Triton X-100, washed once with PBS and twice with Tris-buffered saline (TBS). Cells were incubated with primary antibodies TOM70 (1:200, Proteintech) and p62/SQSTM1 (1:200, Abcam) diluted in TBS overnight at room temperature, and washed with TBS. Cells were incubated in corresponding fluorescent secondary antibodies (1:1000, Invitrogen) for 2 hours in the dark at room temperature, then washed with PBS and stored at 4 °C until imaging using a Keyence BZ-9000 microscope (Keyence; Japan). Color thresholds to visualize green (p62-related) and red (Tom70-related) were set equally in exposure time for collecting images between all samples. A total of 10 similar plane fields from each sample dish were taken for both green and red fluorescence. A total of 4 control DMSO-treated sample dishes and 4 DMB-treated sample dishes were used to obtain 80 fields in total (10 fields per sample dish). Fields taken were randomized among all samples before providing to the blinded scorer. The blinded scorer was instructed to determine the percentage of cells from each field that exhibited a significant number of red-green colocalization events (positive for mitophagy was agreed to be greater than 10 yellow dots in each cell in a field). Example: a field has 10 whole cells visible, 3 of those cells contain 10 or greater yellow colocalization dots and are counted positive. The ten fields corresponding to a particular sample plate were then pooled and an average for percentage cells exhibiting positive mitophagy was determined. The mean of percent averages among the 4 plates in a group was then recorded as shown in the graph Fig. 4d.

### qPCR

Total RNA was isolated from heart lysates following the miRNeasy Mini Kit protocol (Qiagen) with DNAse step. cDNA was prepared according to the iSript cDNA Synthesis Kit (Bio-Rad) using 250 ng of RNA. The cDNA samples followed gene expression analysis according to the Taqman Universal Master Mix II no UNG protocol and Taqman assays (Life Technologies). A set of 4 reference genes (18 S, Gapdh, Eef1a1 and Rpl4) were analyzed by NormFinder^54^, and gene expression followed normalization to the average expression of 18 S and Rpl4 using the 2^−ΔCt^ formula. Data was presented as fold change.

### Statistical analysis

All values are presented as mean ± standard deviation. Standard Student’s *t*-test was used to compare data with 2 groups only. Data with 3 or more groups was analyzed using Analysis of Variance (ANOVA) with Tukey posthoc test. p < 0.05 was considered as statistically significant. All the statistical analysis was performed on GraphPad Prism v.6.

## Supplementary information

Supplementary information.
