# Supplementary material for: Intermittent Use of a Short-Course Glucagon-like Peptide-1 Receptor Agonist Therapy Limits Adverse Cardiac Remodeling via Parkin-dependent Mitochondrial Turnover
Source: Sci Rep. 2020 May 19;10:8284. doi: 10.1038/s41598-020-64924-2 (PMC7237417; doi:10.1038/s41598-020-64924-2)

Intermittent Use of a Short-Course Glucagon-like Peptide-1 Receptor Agonist Therapy Limits Adverse Cardiac Remodeling via Parkin-dependent Mitochondrial Turnover

Juliana de F. Germano, Ph.D.[#,1], Chengqun Huang, Ph.D.[#,1], Jon Sin, Ph.D.[1], Yang Song, Ph.D.[1], Kyle C. Tucker, B.S.[1], David J.R. Taylor, Ph.D.[1], Hannaneh Saadaeijahromi, M.D.[1], Aleksandr Stotland, Ph.D.[1], Honit Piplani, Ph.D.[1], Roberta A. Gottlieb, M.D.[1], Robert M. Mentzer, Jr., M.D.[1], and Allen M. Andres, Ph.D.[*, 1, 2]

[#]These authors contributed equally

*Corresponding Author

[1] Cedars Sinai Medical Center, Smidt Heart Institute

[2] University of California, San Diego, Department of Medicine

Supplementary Figure Legends

Supplementary Figure S1. Body parameters of wild-type mice. Age-matched wild-type (WT) mice underwent PCAL and 2 hours later were given vehicle (50µL DMSO) or DMB (10pmoles/25g) i.p. then three times a week for two weeks (total 6 doses). Mice were sacrificed on day 28 post-MI. (n=7-8 mice/group). **(a)** Mouse weights were recorded on days 0, 3, 7, 14 and 28 and compared between groups; **(b)** Heart weights and tibia length were recorded on day 28 post-MI and heart weight/tibia length ratio were compared between groups; **(c)** Comparison of wet/dry lung ratios; **(d)** Representative images of hearts of vehicle and DMB-treated mice 28 days post-MI; **(e)** Glucose levels of naïve versus WT mice after 3 days of PCAL and treatment with either vehicle or DMB.

Supplementary Figure S2. DMB stimulates fragmentation of mitochondria and decreases mitochondrial membrane potential. (a) Mitochondrial membrane potential was measured with JC-1 ratiometric staining at indicated times after treatment with 1µM DMB. FCCP was used as a positive control to dissipate mitochondrial membrane potential. (n = 18-20 wells/group; n = 4 wells for positive control FCCP group). ANOVA with Tukey posthoc test was used to compare the groups. *DMB *versus* Vehicle; $^{\#}$FCCP *versus* all groups. **(b)** Differentiated H9C2 cells were treated with 1µM DMB for 24 hours and then fixed with 4% PFA. Cells were subsequently immunostained with antibody to TOM70 (green) to highlight mitochondrial structure.

Supplementary Figure S3. Original blots from Figure 2. Age-matched wild-type (WT) mice received vehicle (50µL DMSO) or DMB (10pmoles/25g) i.p.. 1h later, mice received chloroquine (CQ, 10mg/kg i.p.) and were sacrificed 16h later (n = 3/group) for western blot analysis of autophagy and mitophagy markers. Western blot analysis and quantification of autophagy markers p62 **(a)**, LC3-I and -II **(b)** were done in heart whole lysates of WT mice and normalized to the respective Ponceau **(c)**; Western blot analysis and quantification of mitophagy markers Optineurin (OPTN) **(d)**, PINK1 **(e)** and BNIP3 **(f)** in the mitochondrial-enriched fraction of WT mice, with the respective Ponceau **(g)**. Blots were developed with Clarity Western ECL Substrate (Bio-Rad) and imaged using a ChemiDoc XRS and the Image Lab software v 5.0 (Bio-Rad). **Original blots from Figure 3.** Differentiated H9C2 cells were treated with vehicle (DMSO) or 1µM DMB overnight with or without autophagy inhibitor bafilomycin A1 (BafA1, 50nM). Western blot analysis of autophagy markers p62 **(h)**, Beclin-1 **(i)**, APG5 **(j)** and LC3-I and LC3-II **(k)** were performed in the cell whole lysate and normalized to the respective ponceau **(l)** (n = 3 plates/group); **(m,n)** Western blot analysis of p62 in the mitochondria-enriched subcellular fraction of DMSO or DMB-treated cells with or without BafA1 (n = 4 plates/group); **(o,p)** Ponceau from **(m)** and **(n)**, respectively. Blots were developed with Clarity Western ECL Substrate (Bio-Rad) and imaged using a ChemiDoc XRS and the Image Lab software v 5.0 (Bio-Rad). **Original blots from Figure 4.** Age-matched Parkin Knockout (PKO) were treated and sacrificed as the WT mice (described above). Western blot analysis of autophagy markers p62 **(q)** and LC3-I and -II **(r)** were done in heart whole lysates of WT mice and normalized to the respective ponceau **(s)**; Western blot analysis of mitophagy markers BNIP3 **(t)**, Optineurin (OPTN) **(u)** and PINK1 **(v)** in the mitochondrial-enriched fraction of PKO mice, with the respective ponceau **(w)**. Blots were developed with Clarity Western ECL Substrate (Bio-Rad) and imaged using a ChemiDoc XRS and the Image Lab software v 5.0 (Bio-Rad).

Supplementary Methods

Glucose level determination in serum: Whole blood was harvested following mouse sacrifice and heart excision. Samples were at room temperature for 20 minutes then followed centrifugation at 2500 x g for 5 minutes. The serum samples were placed in new tubes and stored at -80°C until use. Glucose levels were assessed using the Autokit Glucose Kit (Wako Diagnostics) following the manufacturer protocol and determined by colorimetric measurement on a SpectraMax M2^{e}.

Immunofluorescence and Microscopy: H9C2 cells were seeded on fibronectin gelatin coated glass bottom microwell dishes (MatTek Corporation) at a density of 2.5 x 10^{3} cells per well, then switched to differentiation media and maintained for five days. Differentiated H9C2 cells were treated with vehicle (DMSO, 0.1% vol) or 1 μM DMB for 24 hours then fixed with 4% paraformaldehyde (Sigma-Aldrich) in phosphate-buffered saline for 10-15 min at room temperature and washed with PBS. Nuclei were stained with Hoechst 33342 (Invitrogen) and rinsed with PBS. Cells were permeabilized with PBS containing 5% horse serum, 5% goat serum and 0.3% Triton X-100, washed once with PBS and twice with Tris-buffered saline (TBS). Cells were incubated with primary antibodies to TOM70 (1:200, Proteintech) to immuno label mitochondria. After rinsing cells three times in TBS, cells were incubated in corresponding fluorescent secondary antibodies (1:1000, Invitrogen) for 2 hours in the dark at room temperature, then washed with PBS and stored at 4°C. Imaging was performed using a Keyence BZ-9000 microscope (Keyence; Osaka, Japan).

Mitochondrial Membrane Potential Assay: Mitochondrial membrane potential was assessed in differentiated H9C2 cardiomyocytes using JC-1 according to manufacturer's instructions. Briefly cells were grown and differentiated on opaque-walled tissue culture-treated 96-well plates with vehicle (DMSO) or 1μM DMB for up to 24 hours. Cells were then stained with 5μM JC-1 in complete differentiation medium at 37°C for 30 min. Cells were rinsed three times with PBS, then imaged immediately using a Spectramax M2 fluorescence plate reader. Membrane potential was expressed as the ratio of red (Ex/Em=540/590nm) to green (Ex/Em= 485/535nm) fluorescence. Negative control cells received 10μM FCCP for 10 minutes before JC-1 addition.

Supplemental Figure S1

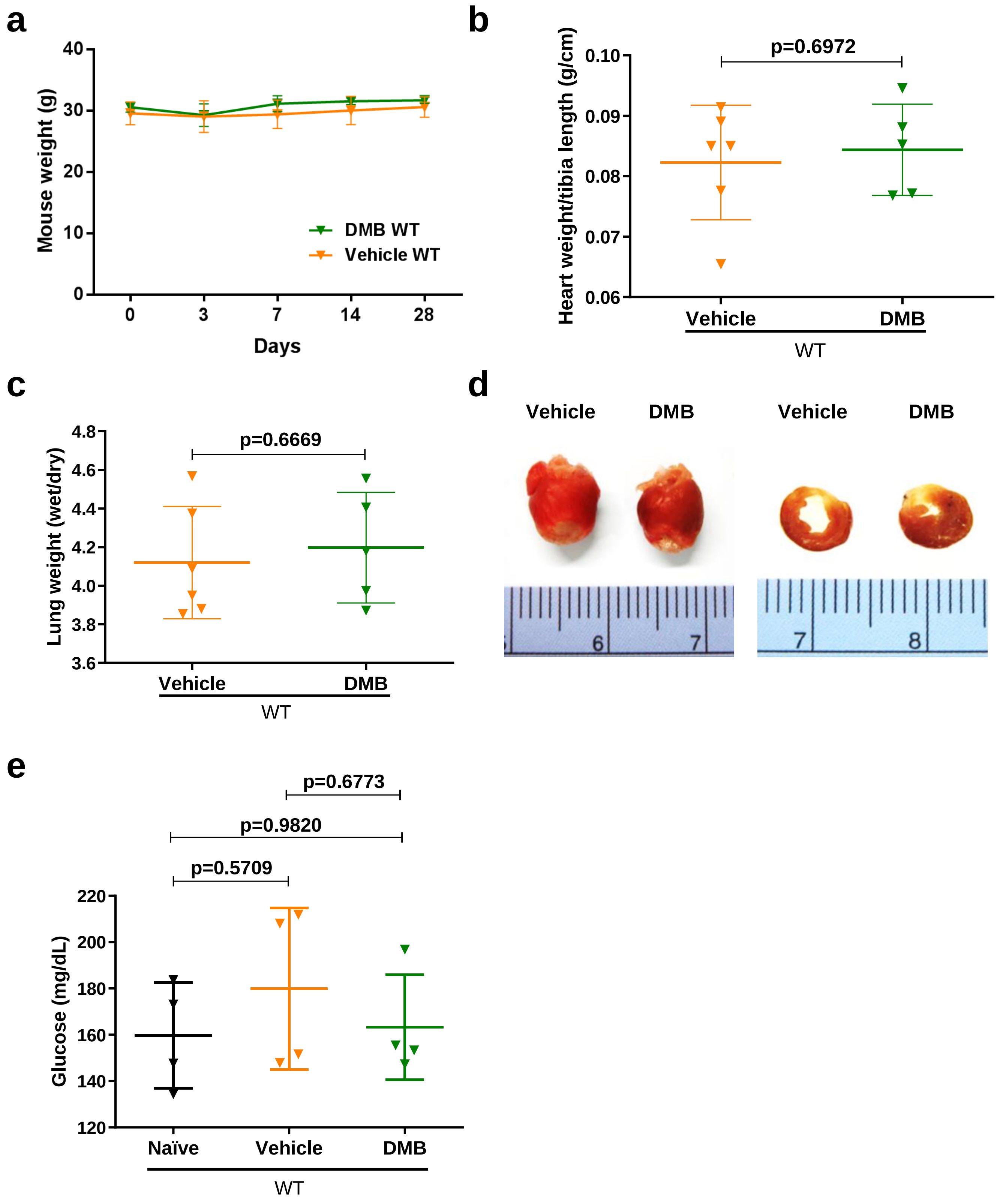

Supplemental Figure S2

a

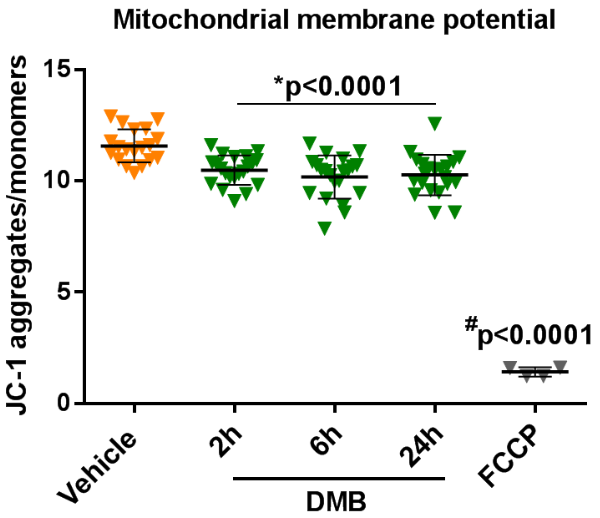

b

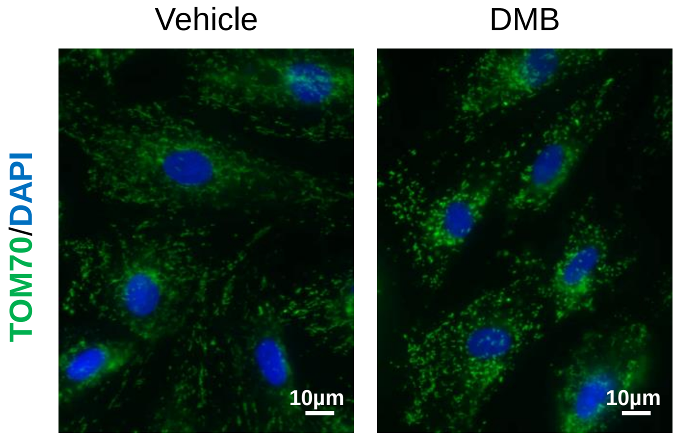

Supplementary Figure 3

Wild-type mice

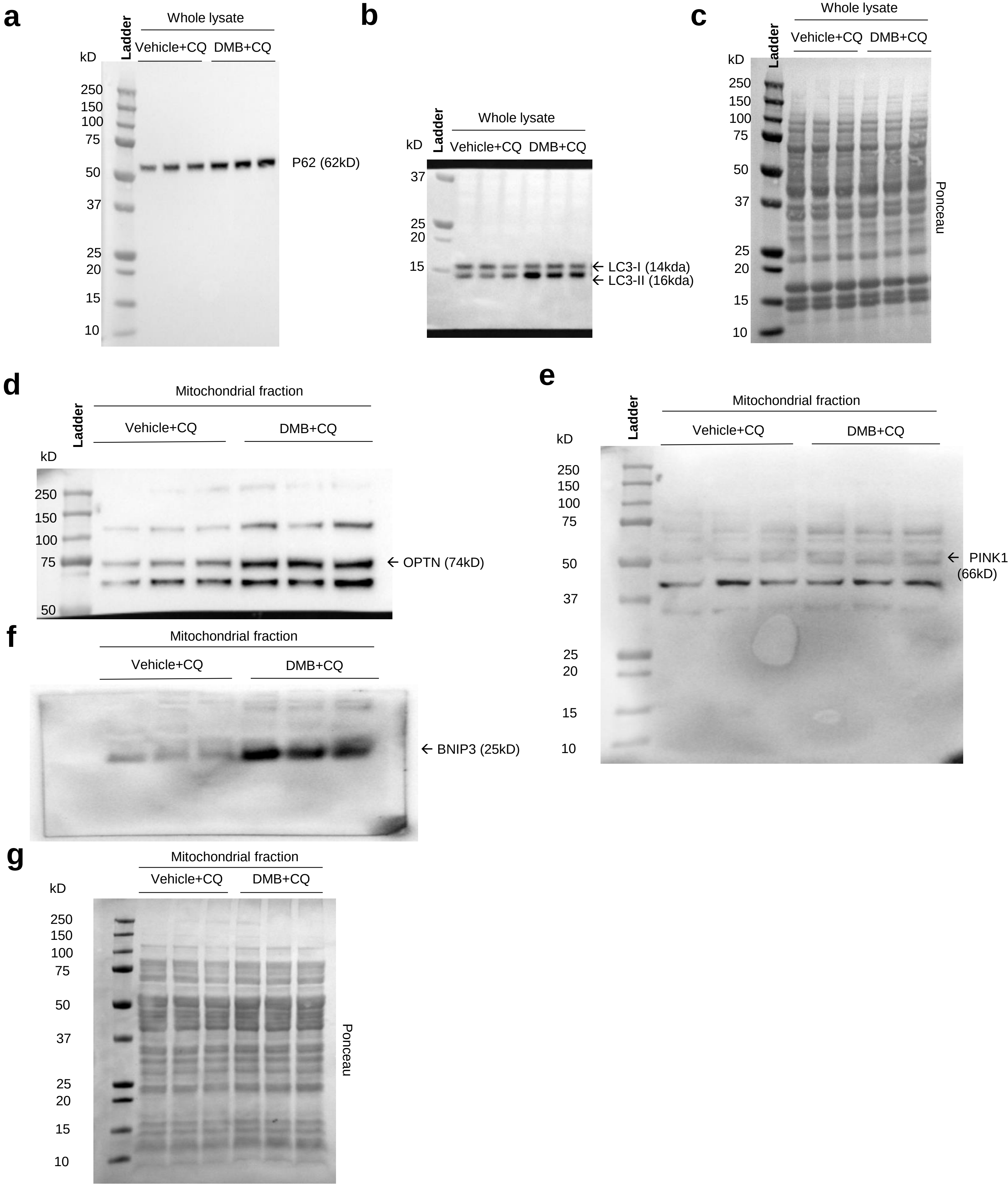

Differentiated H9C2 cells

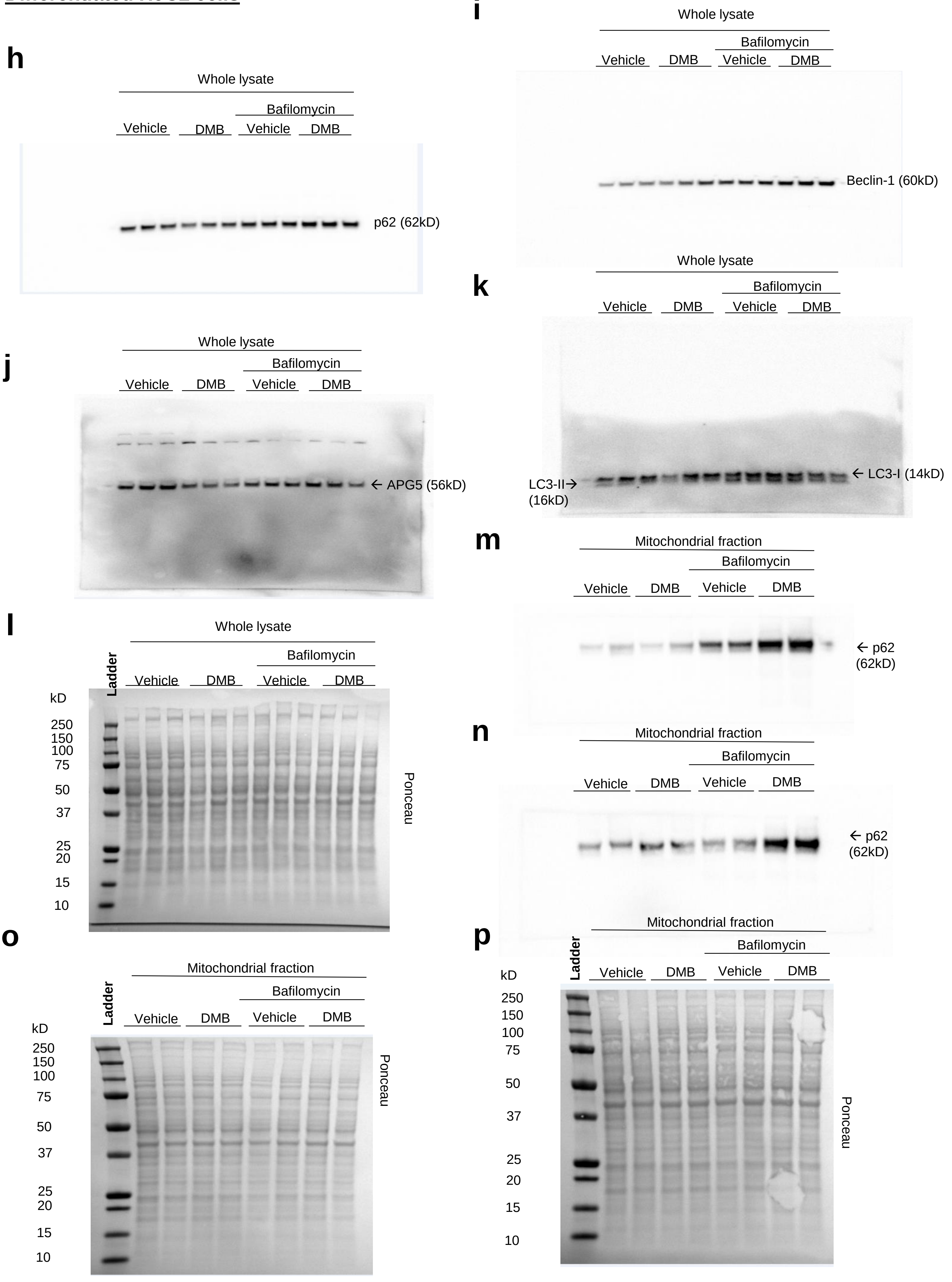

Parkin knockout mice

q

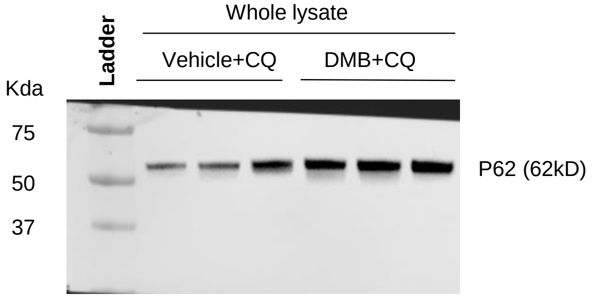

r

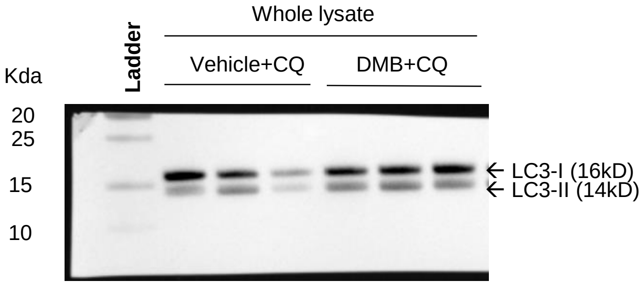

s

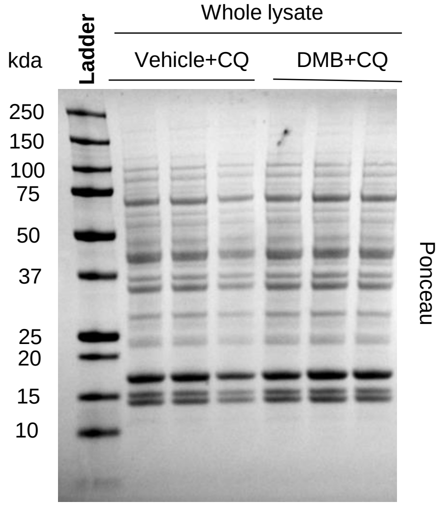

t

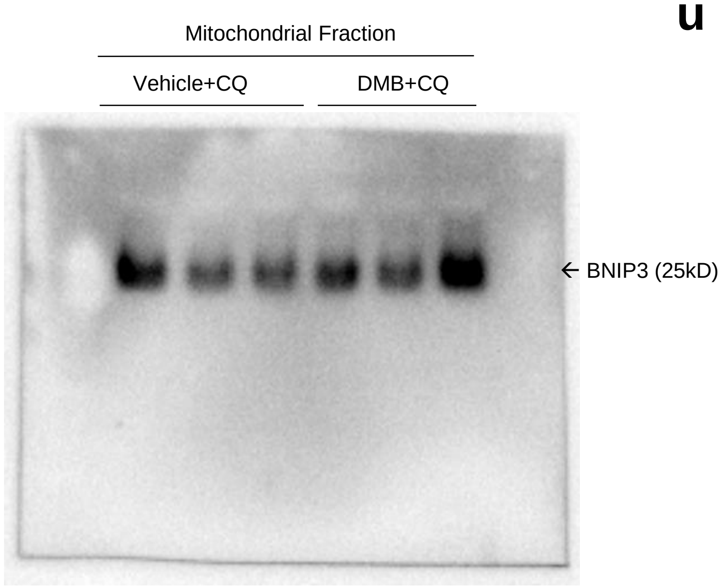

u

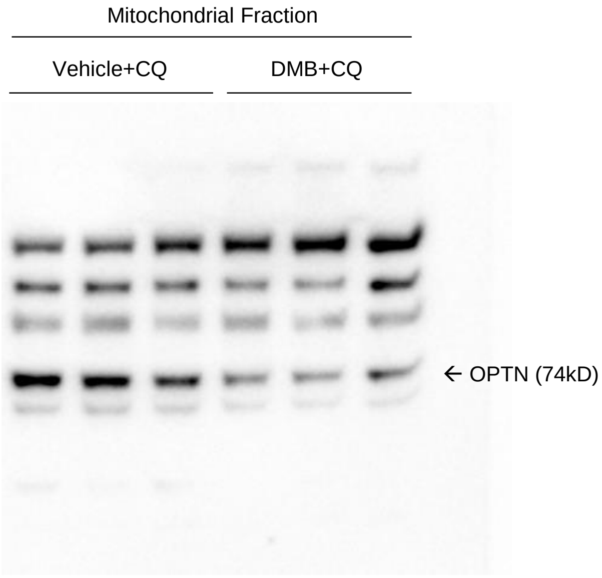

v

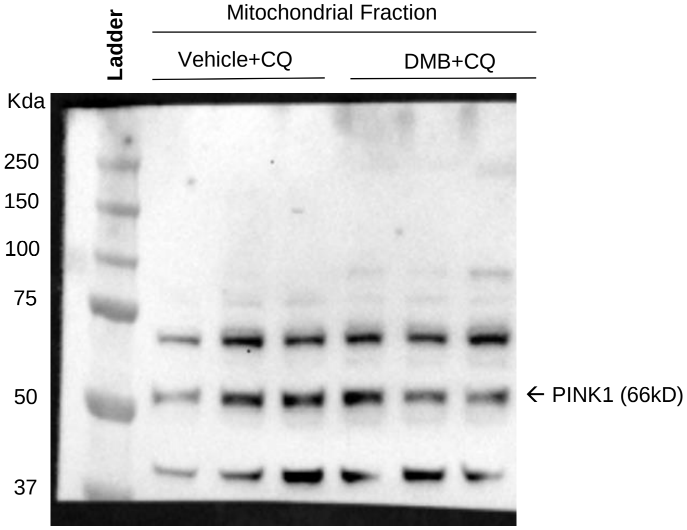

w

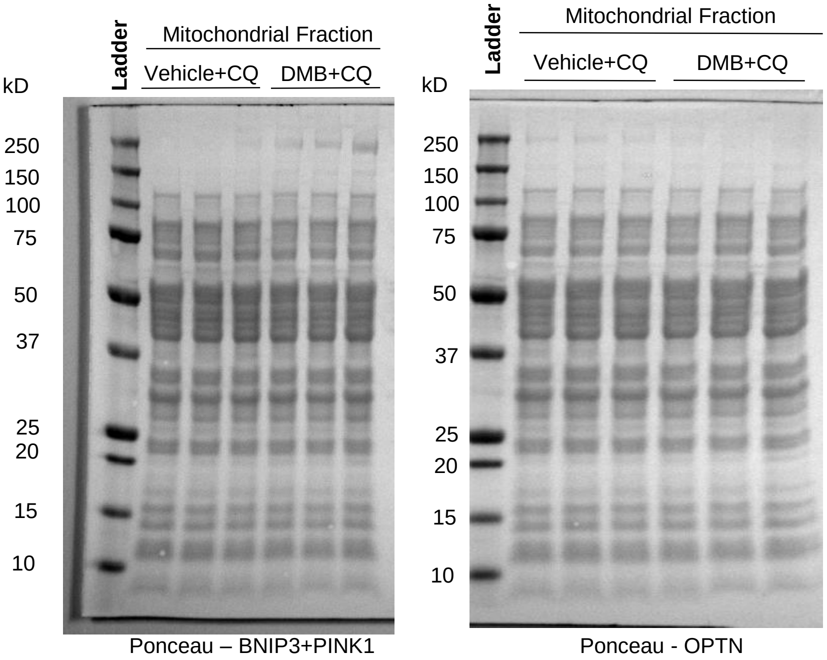

Supplement: Supplementary file 1 — Supplementary information. [file 41598_2020_64924_MOESM1_ESM.pdf]
